# Luteolin improves cardiac dysfunction in heart failure rats by regulating sarcoplasmic reticulum Ca^2+^-ATPase 2a

**DOI:** 10.1038/srep41017

**Published:** 2017-01-23

**Authors:** Wenjing Hu, Tongda Xu, Pei Wu, Defeng Pan, Junhong Chen, Jing Chen, Buchun Zhang, Hong Zhu, Dongye Li

**Affiliations:** 1 Institute of Cardiovascular Disease Research, Xuzhou Medical University, Xuzhou, Jiangsu, 221002, China; 2 Department of Cardiology, Affiliated Hospital of Xuzhou Medical University, Xuzhou, Jiangsu, 221006, China

## Abstract

We previously found that luteolin (Lut) appeared to improve the contractility of cardiomyocytes during ischemia/reperfusion in rats. The enhancement was associated with the alteration in sarcoplasmic reticulum Ca^2+^-ATPase 2a (SERCA2a). This finding prompted us to consider if the mechanism worked in heart failure (HF). We studied the regulation of SERCA2a by Lut in failing cardiomyocytes and intact heart of rats. Improvement of contractility and the mechanisms centered on SERCA2a were studied in isolated cardiomyocytes and intact heart. We found that Lut significantly improved contractility and Ca^2+^ transients, ameliorated expression, activity and stability of SERCA2a and upregulated expression of small ubiquitin-related modifier (SUMO) 1, which is a newfound SERCA2a regulator. Lut also increased phosphorylation of protein kinase B (Akt), phospholaban (PLB) and sumoylation of SERCA2a, specificity protein 1 (Sp1). Transcriptions of SUMO1 and SERCA2a were concurrently increased. Inhibition of posphatidylinositol 3 kinase/Akt (PI3K/Akt) pathway and SERCA2a activity both markedly abolished Lut-induced benefits *in vitro and in vivo*. Lut upregulated the expression ratio of Bcl-2/Bax, caspase-3/cleaved-Caspase3. Meanwhile, Lut ameliorated the myocardium fibrosis of HF. These discoveries provide an important potential therapeutic strategy that Lut targeted SERCA2a SUMOylation related to PI3K/Akt-mediated regulations on rescuing the dysfunction of HF.

Heart failure (HF) is a complex syndrome that results from the deterioration of the cardiac structure and function, characterized by the impaired ability of the ventricle to fill with or eject blood^1^. It is an ultimate common pathway that begins with diverse etiologies, such as hypertension, ischemia, tachycardia, infection, metabolic disorder, and cardiomyopathy, and develops with continual activation of the renin-angiotensin and sympathetic nervous systems. The incidence, prevalence and economic burden of HF are now steadily increasing due to the aging of the population and transition of acute cardiac problems into chronic disorders.

Abnormality Ca^2+^ homeostasis is a universal characteristic of human and experimental HF^2^. Ca^2+^ homeostasis is directly modulated by four proteins: L-type Ca^2+^ channel and Na^+^/Ca^2+^ exchanger (NCX) in cell membrane, Ca^2+^-ATPase and ryanodine receptor type 2 in sarcoplasmic reticulum (SR)^3^. Any abnormality of the expression or activity of the Ca^2+^ handling proteins mentioned above leads to alterations in cardiac contractility.

Sarcoplasmic reticulum Ca^2+^-ATPase 2a (SERCA2a), a principal cardiac form of SERCA, is important in controlling excitation/contraction coupling. SERCA2a’s role in HF has been extensively studied in animal models and human, which have shown that SERCA expression and activity are reduced in failing myocardium^4^. Genetic treatments show that reduction in SERCA2a level results in impaired intracellular Ca^2+^ homeostasis and reduces both systolic and diastolic function^5^,^6^. These results indicate that modulation of SERCA2a is a possible means of regulating cardiomyocytes contractility in HF.

Multiple mechanisms participate in the regulation of SERCA2a function, both its activity and expression. Phospholaban (PLB) is a well-known major regulator of SERCA2a activity. Binding of PLB to SERCA2a inhibits the pump’s affinity for Ca^2+^, whereas phosphorylation of PLB suppresses this inhibition^4^,^7^. SERCA2a activity could also be modulated by post-translational modifications, such as glutathiolation^8^ and nitration^9^. Kho *et al*. showed that sumoylation was a critical post-translational modification in regulating SERCA2a function^10^. Sumoylation is a post-translational modification that can be accomplished by reversibly binding the small ubiquitin-related modifier (SUMO). Sumoylation alters the functional activity of targets by regulating protein stability, protein-DNA binding activity, protein–protein interaction and nucleo-cytoplasmic translocation^11^. Kho *et al*. found that sumoylation of SERCA2a was significantly reduced in cardiac tissue of HF patients. By means of gene therapy, they proved that sumoylation increased the intrinsic activity of SERCA2a ATPase in failing cardiomyocytes, as well as prolonging the life time of SERCA2a. Sumoylation of myocardial targets other than SERCA2a might also contribute to the reversal of cardiac dysfunction^12^. A study by Tilemann *et al*. suggested that sumoylation of Sp1, a recognized transcription factor of SERCA2a in myocardium, resulted in an increase of SERCA2a transcription in the failing heart. Therefore, sumoylation may be an effective target for therapy in HF.

Luteolin (Lut), a widely existing flavonoid in Chinese herbal medicine, enhances the contractility of cardiomyocytes during ischemia/reperfusion (I/R)^13^,^14^, which is mostly related to alteration of SERCA2a function. Our previous study also confirmed that Lut could decrease serum BNP level and partially reverse ventricular remodeling, finally improve cardiac function of HF rats^15^.

In 2007, the clinical trial of SERCA2a gene therapy has been initiated, which was called Calcium Up-regulation by Percutaneous Administration of Gene Therapy in Cardiac Disease (CUPID)^16^. As a first phase 1/2 clinical trial, CUPID 1 got a positive result, CUPID2 were followed conducted in 2012^17^. However, results of CUPID2 trial turned out to be negative; revealing no improvement in recurrent and terminal events in patients received AAV1/SERCA2a, compared to patients with placebo^18^. Reasons for the negatives results of CUPID2 were still not clear. We would discuss the reasons in subsequent section.

Furthermore, most findings of the improvement in cardiac contractile function are attributed to increase SERCA2a protein or augmented PLB phosphorylation, further mechanisms of the regulation have not been fully elucidated. Our another study showed that Lut had cardioprotection against I/R injury by improving the I/R-induced decrease in SERCA2a activity partially via the PI3K/Akt signaling pathway *in vivo*^19^. Therefore in the present study, we expected to explore whether, and if so, how Lut enhances the contractility of failing cardiomyocytes and intact heart in rats.

## Results

### Rats’ survival status and general condition

The 12 weeks later, all rats in Sham groups were alive. 72 rats in AAC group were alive and the rest were died. Among the 17 died AAC rats, 8 died in the first four weeks, none died in the second four weeks, and 9 died in the last four weeks. It was discovered by anatomy of the dead rats that the main cause of the 8 death in the first four weeks was acute HF. The causes of the 9 death in the last four weeks were dyspnea and hydrothorax. The 12 weeks later, when compared with their counterparts in sham group, the survival rats in the AAC group were less active and ate less. The respiratory rate of these rats increased.

### Echocardiography demonstrated the definitely expanded left ventricle and decreased cardiac function of AAC rats induced by pressure overload

Echocardiography on rats subjected to 12 weeks of pressure overload detected the occurrence of HF. In the AAC group, compared with themselves at 0 w, their left ventricles apparently expanded 12 weeks after surgery. More specifically, left ventricular internal diameters of end-diastole (LVIDd) as well as left ventricular internal diameters of end-systole (LVIDs) of 12 w AAC rats were significantly increased (LVIDd: 0.455 ± 0.032 vs 0.783 ± 0.036, p < 0.01, LVIDs: 0.135 ± 0.011 vs 0.558 ± 0.032, *p* < 0.01). Fractional shortening (FS) and ejection fraction (EF) of AAC rats were significantly reduced 12 week after the surgery (FS: 70.19 ± 1.92 vs 28.82 ± 1.44, p < 0.01, EF: 95.28 ± 1.52 vs 61.04 ± 2.23, *p* < 0.01), indicating left ventricle dysfunction.

**Figure 1:**
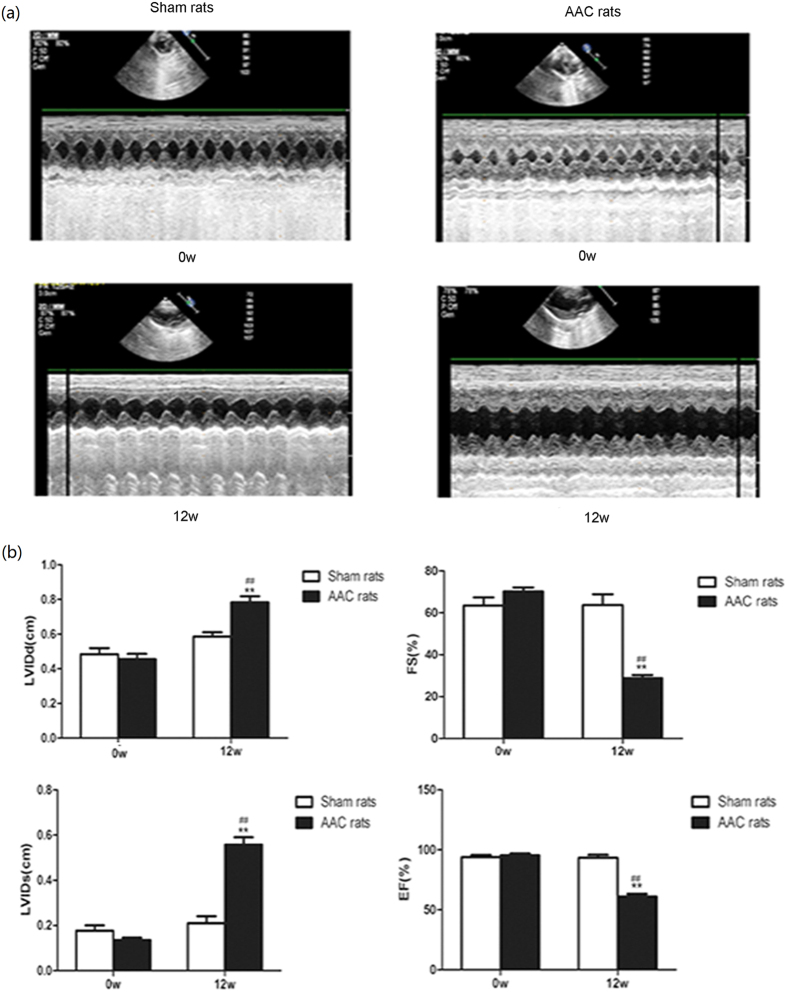
Structural alteration shown by echocardiography in the short-axis view. (**a**) Images of echocardiography. (**b**) Parameters measured by echocardiography. All parameters were measured for at least three successive cardiac cycles. LVIDd: Left ventricular end-diastolic internal diameter. LVIDs: left ventricular end-systolic internal diameter. EF: ejection fraction. FS: fractional shortening. Data are expressed as mean ± SEM. *p < 0.05, **p < 0.01 versus Sham rats 12 w; ^#^*p* < 0.05, ^##^*p* < 0.01 versus AAC rats 0 w.

In comparison with Sham rats 12 w after the surgery, AAC rats had significantly larger ventricles both at diastole and systole of left ventricle. The FS and EF were markedly lower than Sham rats (FS: 63.72 ± 5.15 vs 28.82 ± 1.44, p < 0.01, EF: 93.34 ± 2.47 vs 61.04 ± 2.23, p < 0.01) (Fig. 1).

### Determination of optimal culture time and concentration of Lut

Isolated myocytes from AAC rats twelve weeks after the operation were cultured for 0, 6, 12, 18, 24 and 32 h. Typan blue positive cell (%) was used to determine the optimal culture time. Compared with the 0 h group, no significant increase in the % typan blue positive cell occurred in the 6, 12 and 18 h groups (6 h: 29.65 ± 0.83 vs 23.53 ± 2.99, *p* > 0.05; 12 h: 33.42 ± 3.44 vs 23.53 ± 2.99, *p* > 0.05; 18 h: 32.00 ± 1.55 vs 23.53 ± 2.99, *p* > 0.05), while remarkable increases of blue-stained myocytes were observed in 24 and 30 h groups (24 h: 46.26 ± 2.81 vs 23.53 ± 2.99, *p* < 0.01; 30 h: 68.61 ± 1.76 vs 23.53 ± 2.99, *p* < 0.01) (Supplymentary Fig. a). Thus a culture time of 18 h was chosen for subsequent experiments.

In screening the optimal working concentration, % peak shortening was used to assess the contractility of myocytes at different concentration of Lut, whereas lactate dehydrogenase (LDH) release in supernatant was used to measure the injuries of cultured myocytes treated with different concentrations of Lut. Isolated myocytes from AAC rats were treated with Lut at 0, 4, 8, 16, 32 and 64 μmol L^−1^ % peak shortening improved in a dose-dependent manner at 4, 8, 16 and 32 μmol L^−1^, but was significantly decreased at 64 μmol L^−1^ compared with concentrations of 16 and 32 μmol L^−1^. Among all concentrations of Lut in present experiment, the maximum improvement of cell shortening by Lut was seen at 16 and 32 μmol L^−1^ (Supplymentary Fig. b). LDH release was significantly decreased at 8 and 16 μmol L^−1^ compared with 4 μmol L^−1^ (8 μmol L^−1^: 196.90 ± 17.17 vs 251.50 ± 27.17, *p* < 0.01. 16 μmol L^−1^: 165.30 ± 8.55 vs 251.50 ± 27.17, *p* < 0.01). However, significant increases were seen at 32 and 64 μmol L^−1^ compared with 16 μmol L^−1^ group (32 μmol L^−1^: 235.60 ± 15.54 vs 165.30 ± 8.55, *p* < 0.05. 64 μmol L^−1^: 349.10 ± 8.80 vs 165.30 ± 8.55, *p* < 0.01) (Supplymentary Fig. c). Considering the greater contractility benefits and lower cell injury in the 16 μmol L^−1^ group, this concentration was used for the further experiments.

### Lut improved the contractility and Ca^2+^ transient of failing cardiomyocytes

**Figure 2:**
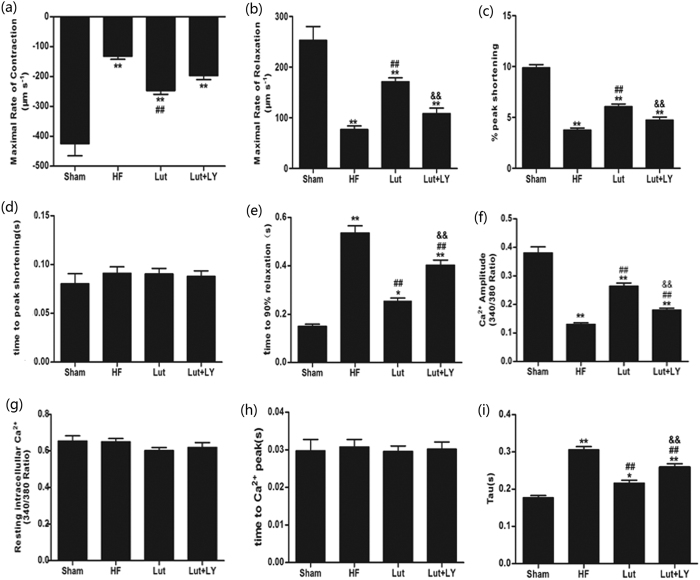
Parameters of cell contractility and Ca^2+^ transient. (**a**,**b**,**c**,**d** and **e**) show the levels of cell contractility parameters. (**f**,**g**,**h** and **i**) show the levels of Ca^2+^ transient parameters. Numbers of myocytes measured in each group listed as follows: n = 14 in Sham group, n = 31 in HF group, n = 31 in Lut group, n = 19 in Lut + LY group. Data are expressed as mean ± SEM. *p < 0.05, **p < 0.01 versus Sham, ^#^*p* < 0.05, ^##^*p* < 0.01 versus HF, ^&^*p* < 0.05, ^&&^*p* < 0.01 versus Lut.

Lut significantly improved the contractility of cardiomyocytes from rats with HF, which showed severely impaired peak shortening (3.75 ± 0.22 vs 9.88 ± 0.27, *p* < 0.01) and reduced maximal rate of contraction (−132.40 ± 9.71 vs −424.50 ± 40.62, *p* < 0.01) and relaxation (76.92 ± 7.05 vs 253.00 ± 27.05, *p* < 0.01), whereas Lut (16 μmol L^−1^) significantly ameliorated contractile dysfunction reflected by both peak shortening (6.07 ± 0.27 vs 3.75 ± 0.22, *p* < 0.01) and maximal rate of contraction (−235.90 ± 14.59 vs −132.40 ± 9.71, *p* < 0.01) and relaxation (170.90 ± 8.11 vs 76.92 ± 7.05, *p* < 0.01). Lut (16 μmol L^−1^) also inhibited the prolonged time to peak shortening (0.0904 ± 0.0056 vs 0.0910 ± 0.0067, *p* > 0.05) and time to 90% relaxation (0.2554 ± 0.0152 vs 0.5375 ± 0.4465, *p* < 0.01) of failing cardiomyocytes, compared to HF. Inhibition of PI3K by LY294002 (LY) markedly reversed the beneficial effect of Lut on contractile function of failing myocytes (*p* < 0.01)(Fig. 2a–e).

No significant difference in resting intracellular Ca^2+^ was detected among all groups. Compared with Sham group, myocytes of HF group had lower Ca^2+^ amplitudes (0.3807 ± 0.0210 vs 0.1302 ± 0.0054, *p* < 0.01) and raised values of Ca^2+^ decay constant (Tau: 0.1769 ± 0.0060 vs 0.3055 ± 0.0085, *p* < 0.01). Reduced Ca^2+^ transients in failing myocytes indicated reduced ability of SR Ca^2+^ clearance. Treatment of Lut significantly raised Ca^2+^ amplitude (0.1302 ± 0.0054 vs 0.2641 ± 0.0111, *p* < 0.01) and acceleration of intracellular Ca^2+^ decay (Tau: 0.3055 ± 0.0085 vs 0.2163 ± 0.0093, *p* < 0.01) compared with the HF group. However, Lut did not elicit any overt effect on time to Ca^2+^ peak (0.0307 ± 0.0020 vs 0.0296 ± 0.0014, *p* > 0.05). LY partly abolished Lut-induced enhancement of intracellular Ca^2+^ transient (Fig. 2f–i).

### Lut activated PI3K/Akt pathway, increased the expression and transcription of SERCA2a and SUMO1 and prompted the phosphorylation of PLB

**Figure 3:**
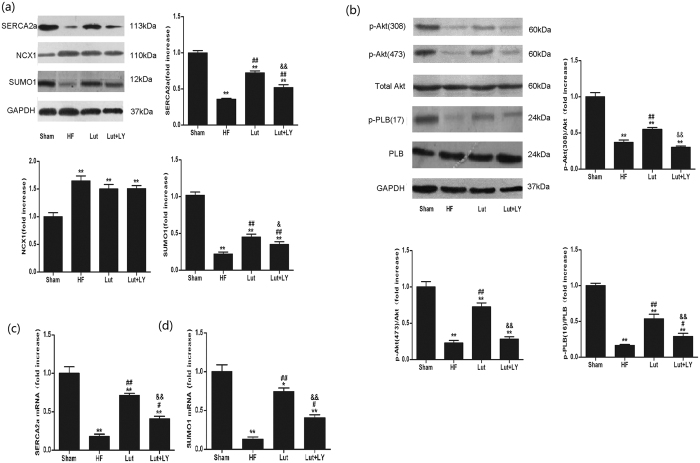
Expression of SERCA2a, NCX1, SUMO1 and phosphorylation of PLB and Akt analyzed by WB, SERCA2a and SUMO1 mRNA analyzed by RT-PCR *in vitro*. (**a**) Expression of SERCA2a, NCX1 and SUMO1. (**b**) Phosphrylation of PLB and Akt. (**c**) Level of SERCA2a mRNA. D. Level of SUMO1 mRNA. n = 3 independent experiment. Results were normalized to GAPDH. Data are expressed as mean ± SEM. *p < 0.05, **p < 0.01 versus Sham, ^#^*p* < 0.05, ^##^*p* < 0.01 versus HF, ^&^*p* < 0.05, ^&&^*p* < 0.01 versus Lut. Results were normalized by GAPDH. n = 3 independent experiment. Data are expressed as mean ± SEM. *p < 0.05, **p < 0.01 versus Sham, ^#^*p* < 0.05, ^##^*p* < 0.01 versus HF, ^&^*p* < 0.05, ^&&^*p* < 0.01 versus Lut.

Western blot analysis showed that Lut significantly triggered the phosphorylation of Akt (both thr 308 and ser 473) compared with the HF group (*p* < 0.01). Phosphorylation of PLB at thr 17 was significantly increased (*p* < 0.01). In parallel with the activation of Akt, SERCA2a and SUMO1 expression increased (*p* < 0.01). However, Lut did not significantly affect the protein level of NCX1 (*p* > 0.05). LY blocked the activation of Akt both at thr 308 and ser 473. Upregulation of SERCA2a and SUMO1 expression was reversed, whereas NCX1 expression was not significantly changed (Fig. 3a,b).

RT-PCR showed that Lut significantly promoted the transcription of SERCA2a and SUMO1 (both *p* < 0.01) compared with HF group. Inhibition of Akt by LY partly reversed the increases of SERCA2a mRNA and SUMO1 mRNA (both *p* < 0.01) (Fig. 3c,d).

### Lut improved the activity and stability of SERCA2a

**Figure 4:**
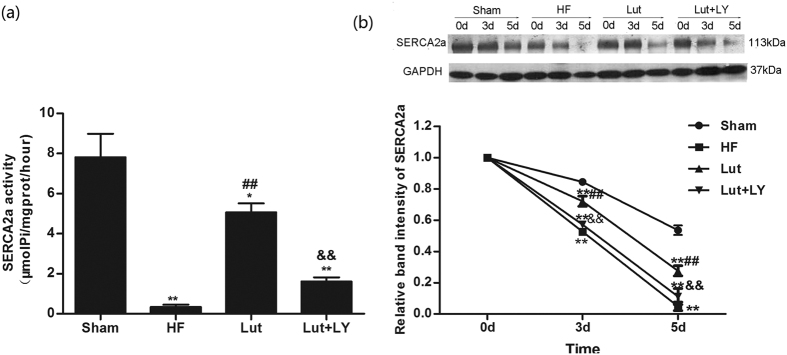
Activity, stability and Sumoylation of SERCA2a *in vitro*. (**a**) Activity of SERCA2a. The data represent three independent experiments. (**b**) Stability of SERCA2a. After protein synthesis was inhibited by cycloheximide, weastern blot analysis was performed to detect the levels of residual SERCA2a at different time points. The quantification data represent the ratio relative to day 0 (n = 3). *p < 0.05, **p < 0.01 versus Sham, ^#^*p* < 0.05, ^##^*p* < 0.01 versus HF, ^&^*p* < 0.05, ^&&^*p* < 0.01 versus Lut.

HF group presented obviously impaired SERCA2a activity. Lut markedly improved SERCA2a Ca^2+^-ATPase activity of failing cardiomyocytes (*p* < 0.01, Fig. 4a). However, inhibition of Akt by LY significantly decreased the improving effect of Lut on SERCA2a activity (*p* < 0.01). The results indicate a partially reversible increase of SERCA2a Ca^2+^-ATPase activity induced by Lut (Fig. 4a).

In cells treated with cycloheximide and cultured for 0, 3, and 5 d, the stability of SERCA2a was measured by its protein concentration. The cells of the HF group had a significantly accelerated degradation of SERCA2a (Fig. 4b). In Lut-treated myocytes, there was an amelioration of SERCA2a stability. Myocytes of the Lut + LY group had clearly reduced SERCA2a levels. Blocking the PI3K/Akt pathway significantly suppressed the stability of SERCA2a (Fig. 4b).

### Lut increased the sumoylation of SERCA2a and Sp1

**Figure 5:**
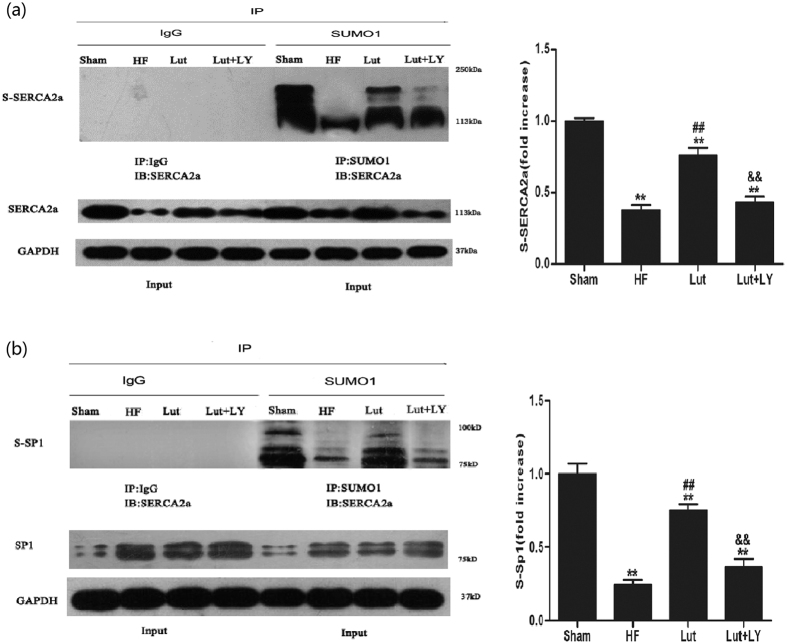
Sumoylation of SERCA2a and Sp1. IP: immunoprecipitation, IB: immunoblotting, S-SERCA2a: sumoylated SERCA2a, S-Sp1: sumoylated Sp1. IgG was used as negative control in immunoprecipitation to exclude interference of primary antibody. (**a**) Level of sumoylated SERA2a. (**b**) Level of sumoylated Sp1. n = 3 independent experiment. Data are expressed as mean ± SEM. *p < 0.05, **p < 0.01 versus Sham, ^#^*p* < 0.05, ^##^*p* < 0.01 versus HF, ^&^*p* < 0.05, ^&&^*p* < 0.01 versus Lut.

Sumoylation of SERCA2a and Sp1 was assessed by their relative band intensities in western blot analysis. Lut significantly increased sumoylation of both SERCA2a and Sp1. Expression of Sp1 was significantly decreased in HF group, whereas Lut treatment increased Sp1 expression. Blocking the PI3K/Akt pathway by LY significantly suppressed the improved sumoylation and protein level of Sp1 (Fig. 5).

### Lut increased expression of Akt and SERCA2a and activity of SERCA2a of HF *in vivo*

**Figure 6:**
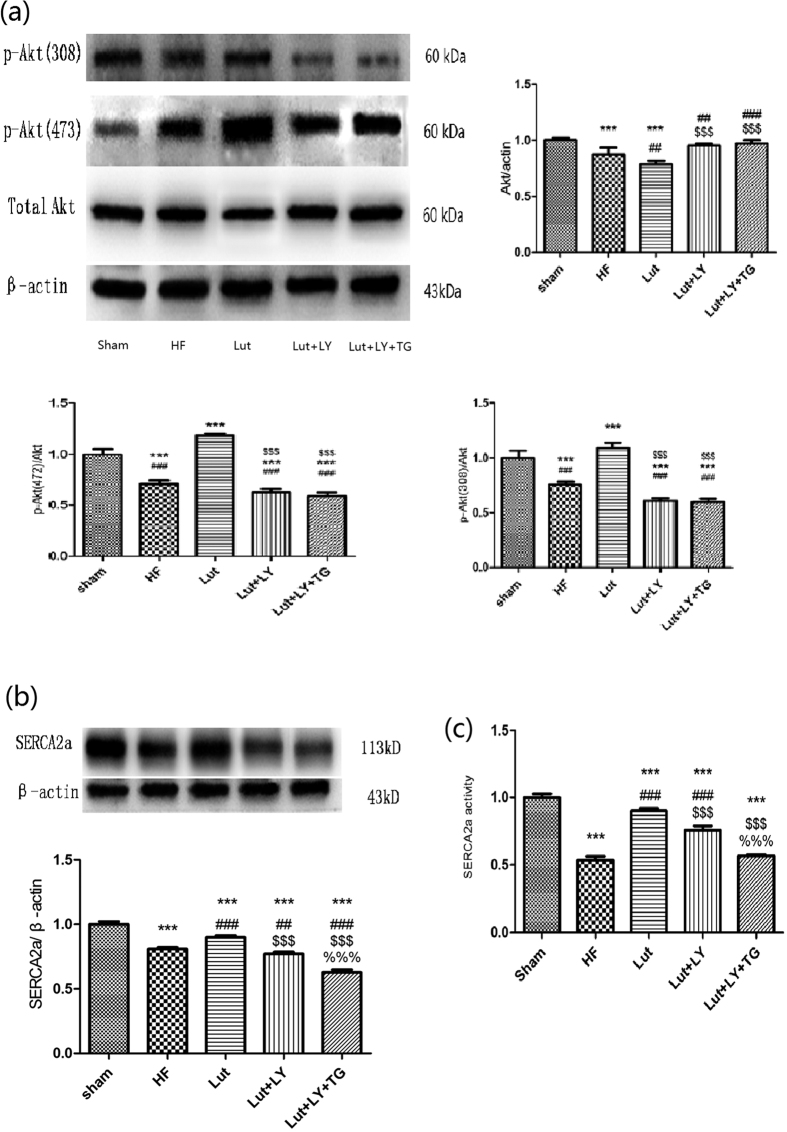
Expression of Akt, SERCA2a and Activity of SERCA2a *in vivo*. (**a**) Expression of Akt and SERCA2a *in vivo*, (**b**) Expression of SERCA2a *in vivo*, (**c**) Activity of SERCA2a *in vivo*. n = 3 independent experiment. Results were normalized to β-actin. Data are expressed as mean ± SEM. ***p < 0.001 versus Sham, ^###^*p* < 0.001 versus HF, ^$$$^*p* < 0.001 versus Lut, ^%%%^*p* < 0.01 versus Lut + LY.

The expression of Akt and SERCA2a were decreased in HF group compared with Sham group (all *p* < 0.01), but Lut improved the expression of Akt and SERCA2a (*p* < 0.01). Meanwhile administration of LY and inhibition of SERCA2a by Thapsigargin (TG) decreased the expression of Akt and SERCA2as (both *p* < 0.01). The similar tendency was also observed in the activity of SERCA2a in HF rats *in vivo (p* < 0.01) (Fig. 6).

### Lut enhanced LV function of rats with HF *in vivo*

**Figure 7:**
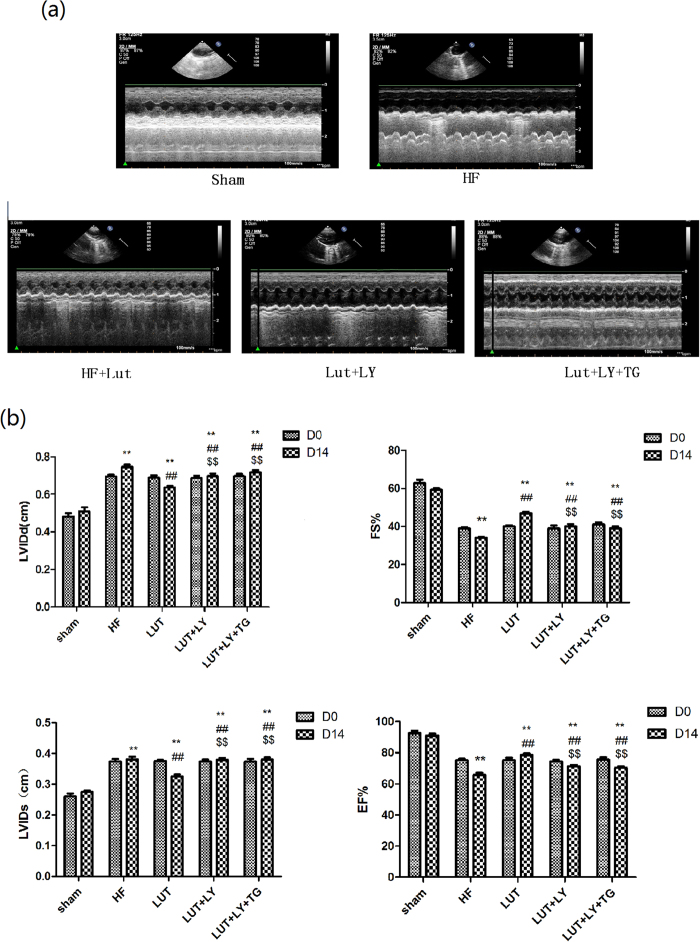
Lut enhanced LV function of heart failure *in vivo*. (**a**) Echocardiography of all groups. (**b**) Measurement of Echocardiography parameters of all groups. LVIDd: left ventricular internal diameters of end-diastole, LVIDs: left ventricular internal diameters of end-systole, FS: fractional shortening, EF: ejection fraction. n = 9 independent experiment. Results were normalized to β-actin. Data are expressed as mean ± SEM. **p < 0.01 versus Sham, ^##^*p* < 0.01 versus HF, ^$$^*p* < 0.01 versus Lut.

LV functions of rats were detected by echocardiography. Our results showed that in HF group, LV function deteriorated compared with Sham group (*p* < *0*.*01*). Administration of Lut could improve LV functions of HF rats. In details, LVIDd and LVIDs in Lut group were significantly decreased compared with HF rats (LVIDd: 0.621 ± 0.031 vs 0.748 ± 0.016 and LVIDs: 0.326 ± 0.076 vs 0.381 ± 0.156, both *p* < *0*.*01*). FS and EF of Lut rats were significantly improved after 14d compared with HF rats (FS: 46.67% ± 1.11% vs 33.67% ± 1.11%; EF: 78.67% ± 1.11% vs 65.67% ± 1.78%, both *p* < *0*.*01*). While administration of LY and LY + TG decreased the improvement of EF and FS value by Lut in HF rat *in vivo* (all *p* < *0*.*01*)(Fig. 7).

### Lut ameliorated myocardium fibrosis induced by HF *in vivo*

**Figure 8:**
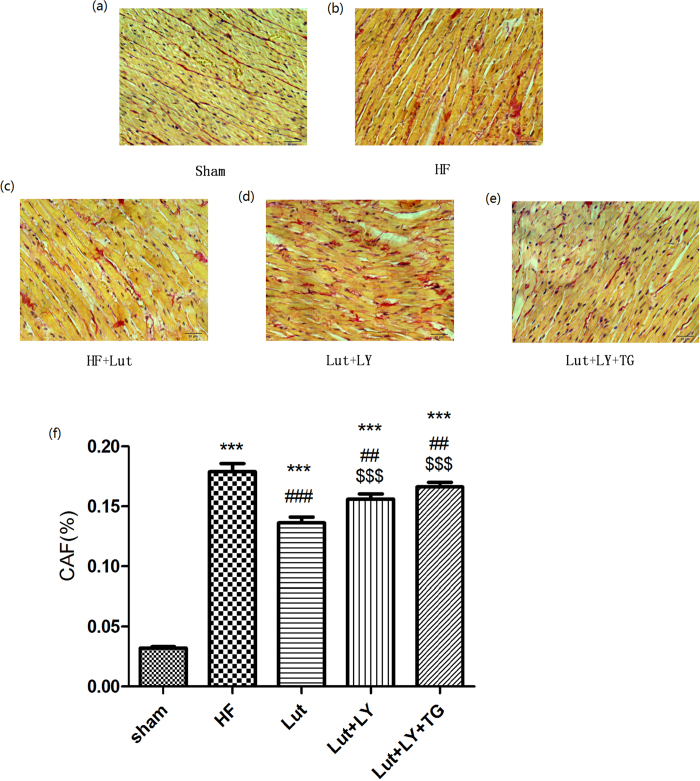
Myocardium section with PSR-staining in all groups. (**a**) sham group. (**b**) HF group. (**c**) Lut group. (**d**) Lut + LY group. (**e**) Lut + LY + TG group. (**f**) Measurement of Collegan area fraction (%). n = 3 independent experiment. Results were normalized to β-actin. Data are expressed as mean ± SEM. bar = 10 μm. ***p < 0.001 versus Sham, ^###^*p* < 0.001 versus HF, ^$$$^*p* < 0.001 versus Lut.

The collagen area fraction (CAF) stained by Picro Sirius Red (PSR) indicated the extent of interstitial fibrosis of myocardium. CAF was increased in HF group compared with Sham group (17.89% ± 2.51% vs 3.21% ± 0.50%, *p* < 0.01). Administration of Lut decreased the CAF content in HF rats (13.62% ± 2.36% vs 17.89% ± 2.51%, *p* < 0.01). While LY and LY + TG were also increased CAF content compared with Lut group *in vivo* (15.60% ± 2.81% and 16.62% ± 3.28% vs 13.62% ± 2.36%, respectively, both *p* < 0.01) (Fig. 8).

### Lut decreased apoptosis of myocardium in rats with HF *in vivo*

**Figure 9:**
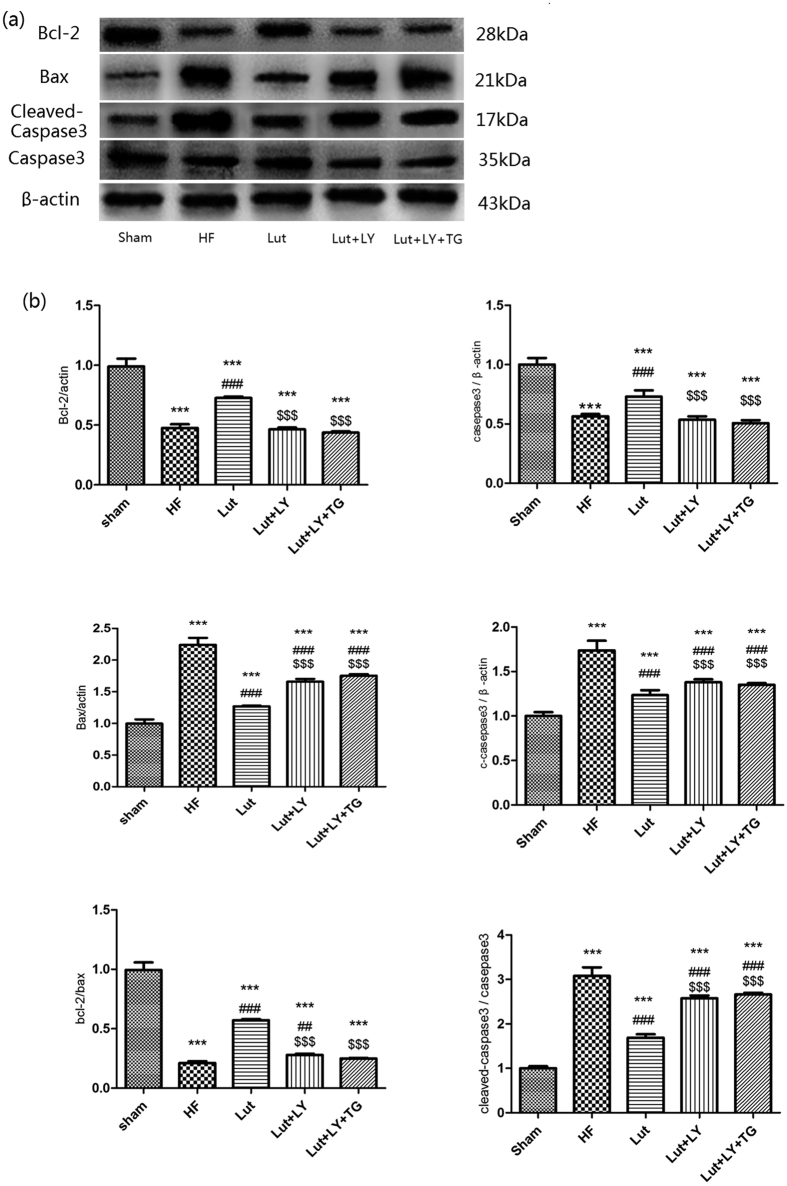
Expression of apoptosis related protein in myocardium *in vivo*. Expression of Bcl-2, Bax, caspase-3, cleaved-caspase-3 in myocardium. n = 3 independent experiment. Results were normalized to β-actin. Data are expressed as mean ± SEM. ***p < 0.001 versus Sham, ^###^*p* < 0.001 versus HF, ^$$$^*p* < 0.001 versus Lut.

The results of western blot showed that in HF group, the expression of Bax was upregulated, but the expression of Bcl-2 and the ratio of Bcl-2/Bax was both downregulated (*p* < 0.001). The results indicated that HF triggered apoptosis of cardiac myocytes compared with Sham group. Treatment of Lut ameliorated the expression of the Bcl-2/Bax compared to HF group (both *p* < 0.001). Administration of LY and LY + TG partly aggravated apoptosis extent compared to Lut group (*p* < 0.001). The change tendency of Cleaved-caspase-3 and caspase-3 were also as well as those of Bax and Bcl-2 (*p* < 0.001) (Fig. 9).

## Discussion

Our previous findings suggested a possible mechanism for Lut-induced enhancement of cardiamyocyte contractility in I/R^13^,^14^. Despite most studies simply relating contractility improvement of Lut to increased SERCA2a expression, we have gained insight into its underlying mechanism by analyzing how enhancement occurs at levels of transcription and post-translational modification. In failing cardiomyocyte and intact heart of rats, Lut not only upregulated the expression of SERCA2a, but also Lut improved ATPase activity and SERCA2a stability. The mechanism involved stages of both transcription and post-translational modification.

During excitation-contraction coupling, action potential leads to the entry of extracellular Ca^2+^ through L-type Ca^2+^ channel. The minor increase of intracellular Ca^2+^ triggers the release of Ca^2+^ from SR via ryanodine receptor type 2. This is called calcium-induced calcium release. After contraction, Ca^2+^ dissociated from troponin complex is mainly reuptaken into SR by SERCA2a (75% of Ca^2+^ removal in human), while a small amount of Ca^2+^ is extruded by NCX (25% of Ca^2+^ removal in human) in sarcolemma^20^. A decrease in SERCA2a (mRNA, protein or activity) is closely correlated with decreased myocardial function. Therefore, restoration of normal level of SERCA2a has been an aim of therapy for HF.

The first-in-human gene therapy trial, *C*alcium *U*pregulation by *P*ercutaneous administration of gene therapy *I*n cardiac *D*iseases (CUPID)^16^,^21^ involved 39 patients with New York Heart Association class III/IV HF, who were treated with SERCA2a adenovirus in 2007. AAV1/SERCA2a improved the symptoms and functional capacity, and reduced clinical events and hospitalization times, CUPID2 enrolled 250 patients with advanced HF in year 2012. However, results of CUPID2 trial turned out to be negative; revealing no improvement in recurrent and terminal events in patients received AAV1/SERCA2a, compared to patients with placebo^18^.

Reasons for the negatives results of CUPID2 were still not clear. The most potential reason might be insufficient delivered-gene expression of myocardium in patients enrolled in AAV1/SERCA2a group of CUPID2. It could be found that The quantity of vector DNA measured in tissue samples was approximate median 43 copies DNA/μg (23 patients, range 10–192) in CUPID 2, much lower than that in CUPID1 (three patients, range 20–561). The factors which contributed to insufficient transgene expression in CUPID2 included extensive presence of AAV1 neutralising antibodies (NAbs)^22^, lower proportion of empty viral capsids^23^, and limitations of intracoronary delivery method^24^. However, number of tissue samples was small, in-depth and detailed analysis of series of CUPID trial should be performed in future.

The cardiac function improved by overexpression of SERCA2a has been extensively demonstrated in a number of experimental animal models and human failing hearts^25^,^26^,^27^,^28^,^29^. SERCA2a is no doubting a potential therapeutic target of HF. Further studies on SERCA2a should concentrate on developing more appropriate vectors and delivery method, promoting efficiency of gene transfer, and most importantly, to explore regulating mechanism of SERCA2a expression and activity.

Our other studies have revealed that Lut protects the myocardium and offers contractile enhancement. Lut upregulates SERCA2a expression through improving of cardiomyocyte contractility in rat I/R model^13^,^14^. However, the mechanism by which Lut affects SERCA2a (mRNA, protein or activity) has not been fully elucidated.

We have found that Lut significantly ameliorates cell shortening and increases the velocity of cell contraction and relaxation. More notably, Lut reduces the relaxation time without a significant effect on contraction time. These initial observations show the difference of Lut benefit between cell contraction and relaxation. Ca^2+^ transient decay time is reduced by Lut, whereas no significant decrease is seen in Ca^2+^ peak time. This suggests that Ca^2+^ uptake rather than Ca^2+^ release is the main process affected by Lut.

Based on this finding, we further detected the expression of SERCA2a and NCX1 (a principal cardiac form of NCX), the 2 main Ca^2+^ handling proteins participating in Ca^2+^ uptake. Treatment of Lut in failing cadiomyocytes upregulated SERCA2a expression, had no significant influenced on NCX1 expression. This suggests that SERCA2a is the major calcium handling protein altered in Lut-treated cardiomyocytes. Increased expression of SERCA2a accelerates Ca^2+^ uptake of SR, reduces the prolonged Ca^2+^ decay time in failing myocytes, and thus directly improves myocyte relaxation. Increased SERCA2a expression restores intracellular Ca^2+^ stores, keeps plenty of Ca^2+^ available for the next contraction, and thus indirectly improves myocyte contraction.

In myocytes isolated from HF rats, Lut upregulates SERCA2a expression, accelerates Ca^2+^ uptake and restores Ca^2+^ stores; improvement of Ca^2+^ cycling also improves contractility. Briefly, alteration of SERCA2a induced by Lut is the primary cause of improved contractility in failing cardiomyocytes.

In the heart, SERCA2a activity controls both the rate of cytosolic Ca^2+^ removal and the degree of SR Ca^2+^ load, representing fundamental determinants of both cardiac relaxation and contraction. Phosphorylation of PLB and direct post-translational modifications of SERCA2a are 2 important ways of regulating SERCA2a activity^30^.

PLB is a small regulatory peptide controlling SERCA2a function. It is a well-recognized molecular switch of SERCA2a: dephosphorylated PLB inhibits SERCA2a activity by decreasing its affinity for Ca^2+^, whereas phosphorylated PLB removes this inhibition^31^.

In HF, expression of PLB appears unchanged, whereas phosphorylation of PLB is significantly decreased. Association of PLB and SERCA2a is disrupted by phosphorylation of PLB at ser10, ser16, or thr17^4^. Kim *et al*.^32^ investigated the underlying mechanism of increased SERCA2a expression by insulin-like growth factor I (IGF-I) in cardiac myocytes isolated from normal adult rats. The expression of SERCA2a increased by IGF-I could be abolished either by the specific PI3K inhibitor or transfection with an adenovirus harboring dnAkt. Moreover, the changes of myocytes overexpressed Akt were observed the same as SERCA2a expression during treatment with IGF-I. The results indicated that SERCA2a expression was directly mediated by the PI3K/Akt pathway in normal rats. Serine/threonine kinase Akt can increase SR Ca^2+^ cycling by direct phosphorylation of PLB at thr17^33^. Therefore, we measured the level of phosphorylated PLB at thr17; Western blot showed a significant increase of phosphorylation of PLB at thr17 in Lut-treated cardiomyocytes of HF rats. These observations indicated that phosphorylation of PLB at thr17 is involved in regulating SERCA2a activity in Lut-treated cardiomyocytes.

SERCA2a activity can also be regulated by direct modulation of the enzyme. Sumoylation is a burgeoning method of investigating post-translational modulation of SERCA2a^34^,^35^. Wang *et al*. found that unbalanced sumoylation and desumoylation is a major factor in congenital HF^36^. Since the sumoylated lysine residues in SERCA2a, K480 and K585, reside in the nucleotide-binding domain that binds ATP, sumoylation might affect the ATPase activity of SERCA2a. Kho *et al*. suggested that sumoylation was essential for normalization of SERCA2a activity and cell contractility^10^, proving that transgene-mediated SUMO1 overexpression rescued pressure overload-induced cardiac dysfunction concomitantly with increased SERCA2a function. Considering the importance of sumyolation in HF, we detected SUMO1 expression in myocytes of the four groups. Western blot analysis showed increased SUMO1 expression in Lut-treated cardiomyocytes. In isolated cardiomyocytes, it was confirmed that sumyolation of SERCA2a increased in Lut-treated failing cardiomyocytes.

In all, our data shows that in Lut-treated failing cardiomyocytes, improvement of SERCA2a activity is due to increased phosphorylation of PLB and sumoylation of SERA2a.

Transcription and protein degradation both affect SERCA2a protein level. Upregulation of transcription has long been seen as a critical mechanism of increasing levels of proteins. Several transcriptional factors have been implicated in the regulation of SERCA2a transcription in HF^37^,^38^. Among these, transcription factor Sp1 is critical in the regulation of SERCA2a gene expression^39^. Sp1 is a ubiquitously expressed transcription factor involved in the regulation of a large number of genes including housekeeping genes as well as actively regulated genes. Recently, it was reported that two proximal Sp1 binding sites within the SERCA2 promoter (Sp1 I and Sp1 III) are responsible for the Sp1 mediated transcriptional modification observed in pressure overload cardiac hypertrophy^40^. In a HF model induced by transverse aortic constriction, consecutive pressure-overload resulted in an increase in Sp1 and a decrease of SERCA2a protein level^12^,^40^. Increase of Sp1 expression in pressure-overload hearts might be a compensatory response that maintains limited transcriptional activity. However, we found the opposite in a changing tendency of Sp1 expression in myocytes of HF group, which indicates that their hearts had lost the control needed to sustain sufficient SERCA2a transcription. Lut treatment improves the expression of Sp1, thus leading to a significant increase in SERCA2a. One of the ways that make Sp1 versatile in transcriptional regulation is its post-transcriptional modification, which alters Sp1 structure and function. Phosphorylation of Sp1 has been studied extensively. Recently, some research have shown that sumoylation of Sp1 might be a critical post-translational modification of Sp1; both SUMO1 and SUMO2 can bind to Sp1 and improve SERCA2a sumoylation, but different SUMOs have different effects on Sp1^41^. Conjugation of SUMO1 to Sp1 enhances transcription, improves DNA binding and increases stability of Sp1. Conjugation of SUMO2 to Sp1 showed distinct effects. In our research, measurement of sumoylated Sp1 showed that Lut upregulated SERCA2a transcription might relate to the increase of sumoylated Sp1. We found that upregulation of SUMO1 expression is accompanied by an increase in sumyolation of Sp1 in Lut-treated cardiomyocytes. Therefore, Lut upregulates SUMO1 expression, increases sumoylation of Sp1 and eventually promotes SERCA2a transcription.

Sumoylation of a target protein often leads to altered protein degradation^42^. Kho *et al*. found that sumoylation of SERCA2a inhibited the poly-ubiquitin-conjugated form of SERCA2a, and suggested that sumoylation competed with ubiquitination of SERCA2a, and prevent its ubiquitin-dependent degradation^10^. To detect whether stability of SERCA2a is improved in failing cardiomyocytes after Lut treatment, we measured residual SERCA2a by western blot, with cycloheximide being employed as an inhibitor of protein synthesis. The higher level of residual SERCA2a in the Lut group indicated improved SERCA2a stability.

Thus we have established that Lut-induced increase in SERCA2a expression is derived from not only upregulated transcription, but increased stability of SERCA2a.

PI3K/Akt pathway regulates a variety of cellular function in cardiomyocytes. Short-term activation of Akt can protect heart from injury, whereas its long-term activation leads to pathological hypertrophy and HF^43^. The PI3K/Akt pathway can improve contractile function by influencing calcium cycling in cardiomyoctes^44^. Lut can activate the PI3K/Akt pathway and upregulate SERCA2a protein, as well as phosphorylation of PLB^13^. Western blot analysis showed that phosphorylation of Thr 308 and Ser 473 in Akt were increased, which suggests that activation of the PI3K/Akt pathway is triggered by Lut. In Lut + LY group; abrogating this improvement further established that PI3K/Akt pathway mediated the regulation of SERCA2A by Lut.

Our results showed that Lut upregulated expression of Bcl-2 and downregulated the expression of Bax as well as caspase-3 and cleaved-caspase-3, decreased apoptosis of failing heart. Meanwhile, the treatment over a certain concentrations range of Lut could inhibit LDH release in the culture supernatant. Combined above results, our results demonstrated that Lut could anti-apoptosis of cardiomyocyte in the model of HF rats.

Lin *et al*.^45^ have established a rat pharmacokinetic model to study pharmacokinetics of Lut *in vivo*. Their results showed that plasma concentration of Lut was rapidly distributed and slowly eliminated after Lut administration in rats. This study also showed that the maximum concentration (Cmax) of Lut was achieved both at 5 min administrated intravenously and orally. But there were differences between intravenously and orally given. The Cmax of Lut 7.47 ± 3.78 cg/mL and 3.07 ± 0.72 cg/mL and the elimination half-life of Lut were 78 ± 14 min and 132 ± 12 min for intravenous and oral administration, respectively. This pharmacokinetic profile indicated Lut was considered to be administered intravenously.

There are several limitations exsisted in the present study. We found that sumoylation of Sp1 increased, and might contribute to the improvement of SERCA2a transcription. Gong *et al*. suggested that different SUMOs display different effects on Sp1^41^. Conjugation of SUMO1 to Sp1 enhances transcriptional activity, improves DNA binding ability, and increases Sp1 stability. Conjugation of SUMO2 to Sp1 has distinct effects. In the present study, we have proved that SUMO1 expression is increased and participates in Sp1sumoylation, but further research is needed to detect whether SUMO2 is involved in these effects in Lut-treated cardiomyocytes. In this study, we confirmed that myocardial fibrosis in HF has been ameliorated by administration of Lut and partly reversed after administration of LY and LY + TG. These results indicated that Lut could inhibit the progress of fibrosis of HF, and the mechanism was partly mediated by Akt and SERCA2a.

## Conclusion

In failing cardiomyocytes and intact heart, Lut activates the PI3K/Akt pathway, promotes the phosphorylation of PLB and the sumoylation both of SERCA2a and Sp1. This improves the protein level and ATPase activity of SERCA2a. The above changes eventually lead to improvement of myocardium and myocyte dysfunction in HF rats.

## Method

### Animal models

Ninety-one male Sprague-Dawley rats weighing 180–250 g were obtained from the Experimental Animal Centre of Xuzhou Medical University. The studies were approved by Animal Ethics Committee of the Xuzhou Medical University, and conformed to the Guide for the Care and Use of Laboratory Animals published by the US National Institutes of Health (NIH Publication No. 85–23, revised 1996) and ARRIVE guidelines. Rats were randomly selected for abdominal aortic separation (Sham group, n = 19) and abdominal aortic constriction operation (AAC group, n = 72). Pressure-overload was induced by abdominal aortic constriction above the left kidney. Rats were anesthetized with 3% pentobarbital sodium (30 mg kg-1) and performed an operation, the abdominal aorta was constricted by a silver clip (Xinhua surgical instrument company, Shandong, China) with an inside diameter of 0.7 mm. The sham group underwent a similar procedure without constriction.

Twelve weeks later, the rats were anesthetized with 3% pentobarbital sodium (30 mg kg^−1^) and examined by transthoracic echocardiography using an IE33 cardiac ultrasound instrument (Philips, Holland), the probe being Model S12 with a frequency of 12 MHZ. Two-dimensional and M-mode images were obtained in the short-axis view. LVIDd and LVIDs were measured for at least three successive cardiac cycles. The EF and FS were then calculated.

Twelve weeks later, rats including sham group and HF group (Only rats in AAC group with FS < 50% were determined as being in HF and suitable for further study^46^) was subsequently preceded into study *in vitro* and *in vivo*.

### Isolation of cardiomyocytes

Isolated heart was perfused for 5 min with Ca^2+^-free buffer before being switched to the same perfusion buffer containing 0.08% collagenase type II (Gibco Inc, Grand Island, USA), 0.01% bovine serum albumin (Amersco, USA) and 50 mmol L^−1^ CaCl_2_. After 25 min of enzymatic isolation, hearts were removed from the cannula, the left ventricles were cut into small pieces and resuspended in Krebs-bicarbonate solution (mmol L^−1^: 15 NaCl, 85 KCl, 30 KH_2_PO_4_, 5 MgSO_4_, 5 sodium pyruvate, 5 creatine, 20 taurine, 2 L-glutamic acid, 20 HEPES, 20 glucose, 0.2 CaCl_2_ and 0.5 EGTA, gassed with 100% O_2_ at 37 °C, pH 7.2). Isolated cardiomyocytes from sham operated rats were cultured as Sham (Sham, n = 10), whereas isolated cardiomyocytes from AAC rats were divided into 3 groups: HF group (HF, n = 15), Lut group (Lut, n = 15), Lut + LY group (Lut + LY, n = 15). All the groups were cultured in Dulbecco’s minimal essential medium (DMEM, Gibco Inc, Grand Island, USA) containing 1% penicillin- streptomycin (Beyotime Institute of Biotechnology, Shanghai, China) at a density of 2 × 10^4^ cells/well in 12-well culture dishes and placed in a carbon dioxide incubator (Heraeus, Germany) with atmosphere of 95% O_2_ and 5% CO_2_ at 37 °C.

### Animal groups *in vivo*

Rats in AAC group confirmed as being in with HF were randomly divided into four groups, including HF group (n = 9), HF + Lut group (Lut group, n = 9), HF + Lut + LY group (Lut + LY group, n = 9) and HF + Lut + LY + TG group (Lut + LY + TG group, n = 9). LY and TG^47^ were used as inhibor of Akt and SERCA2a respectively. Sham group and HF group were fed for 14d without any treatment. Lut group were fed with Lut administration (10 ug/Kg/d, ip)^48^ for 14d. Lut + LY group were fed with Lut administration (10 ug/Kg/d, ip) and LY administration (0.3 mg/Kg/d, ip)^19^ for 14d. Lut + LY + TG group were fed with Lut administration (10 ug/Kg/d, ip) and LY administration (0.3 mg/Kg/d, ip) for 14d, and subjected to SERCA2a inhibitor TG (1 mg/kg/d, ip) on the twelfth day^47^.

### Trypan blue staining

Cell viability of each group was assessed by trypan blue staining. 100 ul 0.4% trypan blue was added to 1 ml cell suspension. After 3–5 min of equilibration, the percentage of blue cells was quantified by microscopy. At least 300 cells per well were counted. Trypan blue positive fraction = number of trypan blue myocytes/total number of myocytes counted × 100%.

### Measurement of contractility

Mechanical properties of ventricular myocytes were measured using an IonOptix Myocam system (IonOptix Corp., Milton, MA, USA). Cultured cells were suspended in the Ca^2+^-free Tyrode’s solution, and Ca^2+^ was slowly added until Ca^2+^ reached a final concentration of 1.8 mmol L^−1^. Cardiomyocytes placed in a chamber on the stage of an inverted microscope were stimulated at 0.5 Hz for 3 ms duration by a pair of platinum wires placed on the opposite sides of the chamber. Electrical pulses were given with an isolated stimulator (FHC Inc., Bowdoinham, ME, USA). Parameters measured were: % peak shortening, maximal velocities of contraction and relaxation, time to peak shortening and time to 90% relaxation.

### Measurement of intracellular Ca^2+^ transient

Myocytes were loaded with fura-2/AM (1 μM, Dojindo Labratories, Japan) for 10 min at room temperature, and imaged with an Olympus IX-70 Fluor 40X oil objective. A dual-excitation fluorescence photomultiplier system was used to determine the intracellular calcium. Myocytes were exposed to light emitted by a 75 W lamp and passed through either a 360 or 380 nm filter during stimulation at 0.5 Hz. Fluorescence was monitored and analyzed by a Soft-edge software (IonOptix Corp., Milton, MA, USA), and qualitative changes of intracellular Ca^2+^ concentration were inferred from the ratio of fura-2 fluorescence intensity at the 2 wavelengths. Other parameters include resting intracellular Ca^2+^, Ca^2+^ amplitude, time to Ca^2+^ peak and Ca^2+^ transient decay time (reflected by the value of Tau).

### Measurement of SERCA2a acvtivity

SR was prepared according to the method of Pande *et al*.^49^. Cultured cells or iced myocardium tissue were transferred to ice-cold homogenizing medium containing 50 mM Na_2_HPO_4_, 10 mM Na_2_EDTA, and 25 mM NaF, pH 7.4. They were homogenized in 1 ml homogenizing medium using Pro200 (Pro Scientific Inc, USA) at its middle power 6 times each of 15 s. The homogenate was sedimented twice for 20 min at 14,000× g at 4 °C. The supernatant was recentrifuged at 45,000× g for 30 min. The sediment obtained consisting of crude membrane vesicles (SR) was suspended in storage buffer consisting of 30 mM histidine, 0.25 M sucrose, 10 mM EDTA, and 10 mM NaF, pH 7.4 and stored at −80 °C.

SERCA2a activity was determined with Ca^2+^ ATPase assay kit (Nanjing Jiancheng Bioengineering Institute, Nanjing, China) by measuring the inorganic phosphate liberated from ATP hydrolysis. SR membranes were added to the reaction mixture containing 50 mM histidine, 3 mM MgCl_2_, 0.1 M KCl, 5 mM sodium azide, 3 mM ATP and 0.05 mM CaCl_2_ (pH 7.0), and incubated for 10 min at 37 °C. The reaction was initiated by addition of ATP. Ca^2+^ ATPase activity was the amount of inhibition caused by 1 mM EGTA. ATP hydrolysis occurring in the absence of Ca^2+^ was subtracted to determine the activity of Ca^2+^-stimulated ATPase. Mitochondrial contamination was assessed by the activity of azide sensitive ATPase, *i*.*e*. activity inhibited by 5 mM sodium azide.

### Assessment of SERCA2a stability

After the preceding manipulation of the cultures, the media containing Lut (Sigma-Aldrich, St. Louis, USA) or LY (Sigma-Aldrich, St. Louis, USA) was discarded from suspension and replaced with culture media containing 100 μg ml^−1^ cycloheximide (Solarbio, Beijing, China). Protein of cells from each group was extracted on days 0, 3 and 5 after cycloheximide treatment. The remaining SERCA2a was detected by western blot analysis.

### Assessment of sumoylation

Cells were lysed in ice-cold lysis buffer (NP-40: PMSF = 100:1, Beyotime Institute of Biotechnology, Shanghai, China). Lysates were cleared by centrifugation at 4 °C 15000 g for 10 min before being precleared by 1.0 μg rabbit Ig G (ZSGB-Bio, Beijing, China) and 20 μl Protein A/G plus–agarose (Santa Cruz Biotechnology, Santa Cruz, CA). Cell lysates were equally divided into 3 EP tube, then the first was left without treatment, the second was added with rabbit IgG and the third was added with SUMO1 antibody (Santa Cruz Biotechnology, Santa Cruz, CA), respectively. The mixtures were incubated at 4 °C overnight and followed by addition of protein A/G plus-agarose and incubation overnight. After centrifugation at 1,000 g for 5 min, the sediment was resuspended in phosphate buffer saline (PBS) and boiled for 3 min. The supernatant was stored at −20 °C. These immunocomplexes were resolved on SDS-PAGE, and western blot analysis was used with the SERCA2a antibody (Abcam, Cambridge, USA) or Sp 1 antibody (Santa Cruz Biotechnology, Santa Cruz, CA).

### Real-time PCR

Total RNA were extracted from cultured cells using TRIzol. The concentration of total RNA was determined by spectrophotometry. Purity of RNA was confirmed by an optical density 260/280 nm ratio between 1.8 and 2.0. Total RNA was reverse-transcribed to cDNA at 70 °C for 5 min, and cooled on ice. A first-strand cDNA synthesis kit (Tiangen, Beijing, China) was used at 42 °C for 50 min, followed by a step of 95 °C for 5 min to terminate the reaction.

Real time PCR analysis was carried out using SYBR Green qPCR Master mix (Tiangen, Beijing, China), the reaction was run in a total volume of 20 μl containing 1 μl cDNA as a template. Thermal cycling was by the methods of Wu^50^. The fluorescence of the SYBR green dye was plotted as a function of PCR cycle number. To confirm the specificity of amplification, the PCR products from each primer pair were analyzed by their melting curves. The Δ CT values (Ct = cycle threshold value) of the target genes (SERCA2a and SUMO1) were calculated by subtracting the values of the experimental group from the housekeeping gene (GAPDH) values. The 2-∆ (∆Ct) method was used to calculate the relative expression of SERCA2a and SUMO1 mRNA. The primer sequences designed with online tools were as follows:
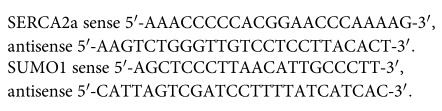


### Western blot analysis

The protein expression was examined by western blot analysis as previously described^16^,^19^. Total protein was harvested from cultured cells or myocardium tissue. Briefly, samples were separated on 8–12% sodium dodecyl sulfate polyacrylamide gel electrophoresis (SDS–PAGE), and then transferred to a nitrocellulose membrane by electroblotting. The membranes were blocked in 5% bovine serum albumin for 3 h, and then incubated with first antibody anti-SERCA2a, anti-NCX1, anti-SUMO1, anti-p-PLB, anti-PLB (Santa Cruz Biotechnology, Santa Cruz, CA, USA), anti-Akt and anti-phospho-Akt (Cell Signaling technology, CST, USA). After overnight incubated at 4 °C, the membranes were then washed and incubated with second antibodies for another 1 h at room temperature. Bands were visualized by the use of a super-western sensitivity chemiluminescence detection system (Tanon Imaging System, Tanon, China). Autoradiographs were quantitated by densitometry (Tanon Imaging System, Tanon, China). GAPDH or β-actin was the internal control for protein normalization.

The expressions of apoptosis protein including Bcl-2, Bax, caspase-3 and cleaved-caspase-3 were also detected by western blot analysis. Anti-Bcl-2, anti-Bax, anti-caspase-3 and anti-cleaved-caspase-3 were brought from Santa Cruz Biotechnology (Santa Cruz, CA, USA).

### Histological analysis and Cardiac fibrosis assessment

Heart were fixed with formalin for 48 h, then dehydrated overnight and embedded in paraffin for cutting. Sections (4 mm thick) were stained with Hematoxylin and Eosin (HE) or PSR reagent (Vicmed, Jiangsu, China). PSR staining was applied for detecting collagen to determine the degree of cardiac fibrosis. Six fields of PSR staining were randomly selected, and PSR-stained fibrosis area was evaluated in comparison to total myocardium area with the use of Image Pro-plus 5.0 software for calculating the percent of the cardiac collagen fraction.

### Statistical analysis

Values are expressed as mean ± S.E.M. *Unpaired t*-test was used for the differences of echocardiography parameters between 0 and 12 w after the operation of Sham or AAC operated rats, and also applied for evaluating the differences of cardiac function between two groups at the same time-point. One-way or two-way ANOVA was used across all groups, followed by a Bonferroni post-hoc correction for all group comparisons. *p* < 0.05 was considered statistically significant. All analyses were performed with GraphPad Prism (GraphPad Software, San Diego, CA, USA). Each experiment was repeated at least three times.

## Additional Information

**How to cite this article**: Hu, W. *et al*. Luteolin improves cardiac dysfunction in heart failure rats by regulating sarcoplasmic reticulum Ca^2+^-ATPase 2a. *Sci. Rep.* **7**, 41017; doi: 10.1038/srep41017 (2017).

**Publisher's note:** Springer Nature remains neutral with regard to jurisdictional claims in published maps and institutional affiliations.

## Supplementary Material

Supplementary Information
