# Supplementary material for: Luteolin improves cardiac dysfunction in heart failure rats by regulating sarcoplasmic reticulum Ca2+-ATPase 2a
Source: Sci Rep. 2017 Jan 23;7:41017. doi: 10.1038/srep41017 (PMC5253630; doi:10.1038/srep41017)
Supplement: Supplementary Information [file srep41017-s1.doc]

Luteolin improves cardiac dysfunction in heart failure rats

by regulating sarcoplasmic reticulum Ca^2+^-ATPase 2a

Wenjing Hu^1^, Tongda Xu^2^, Pei Wu^1^, Defeng Pan^1^, Junhong Chen^2^, Jing Chen^2^, Buchun Zhang^1^, Hong Zhu^2*^, Dongye Li^1*^

^1^Institute of Cardiovascular Disease Research, Xuzhou Medical University, Xuzhou, Jiangsu, 221002, China;

^2^ Department of Cardiology, Affiliated Hospital of Xuzhou Medical University, Xuzhou, Jiangsu, 221006, China.


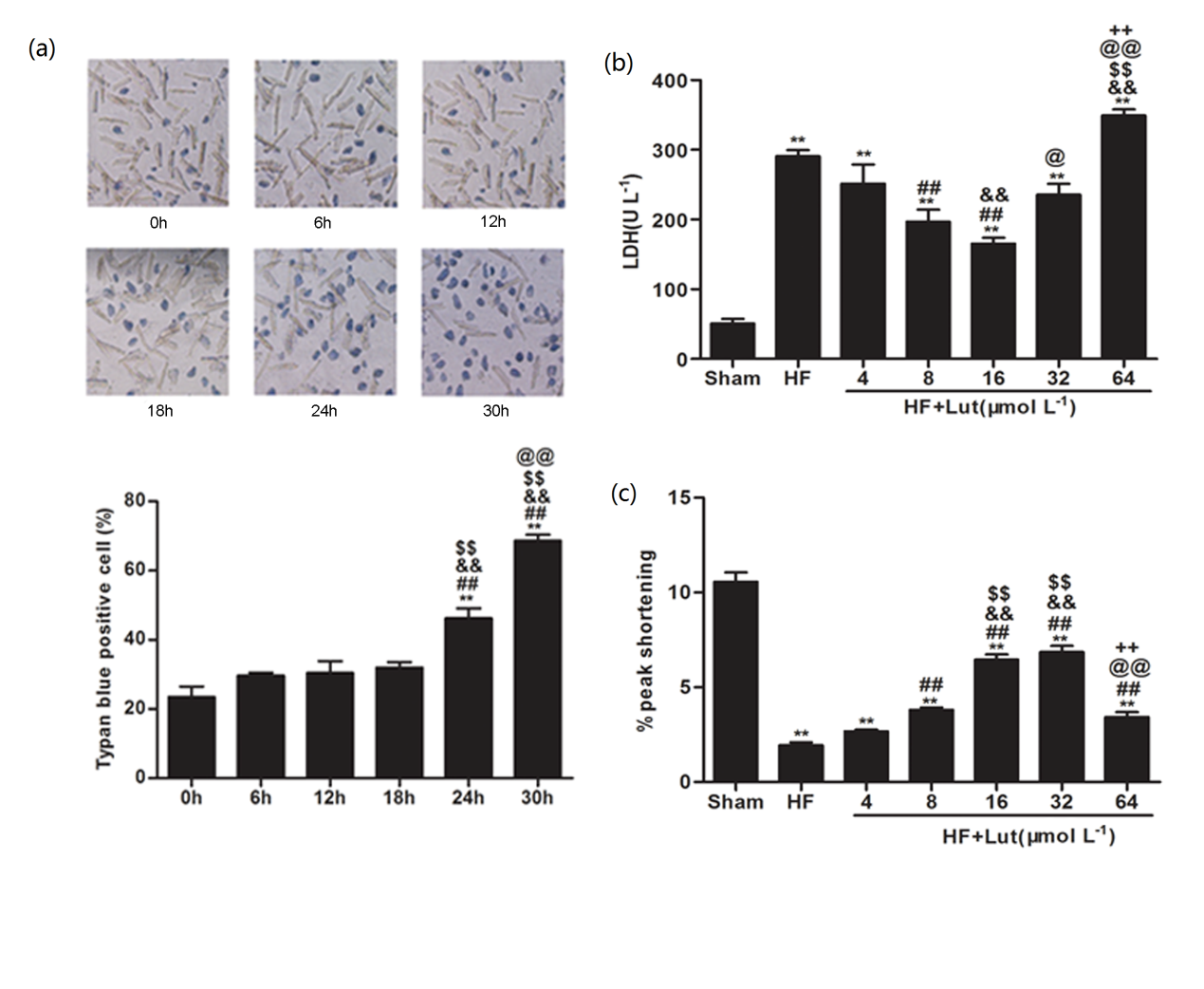


Supplementary Figure. Screening of optimal cultured time and concentration of Lut. (a). Trypan blue positive fraction of cultured cardiomyocytes. In each group, a total of at least 300 cells were counted. (b). LDH release and cell shortening in cultured cardiomyocytes. Results of LDH measurement are representative of three dependent experiments. In peak shortening measurement, numbers of myocytes measured in each group listed as follows: n=10 in Sham group, n=11 in HF group, n=11 in 4 μmol L-1 group, n=11 in 8 μmol L-1 group, n=11 in 16 μmol L-1 group, n=11 in 32 μmol L-1 group, n=11 in 64 μmol L-1 group. Data are expressed as mean ± SEM. **p*<0.05,***p*<0.01 versus Sham, #*p*<0.05, ##*p*<0.01 versus HF. &*p*<0.05, &&*p*<0.01 versus HF+Lut (4μmol L-1), $*p*<0.05, $$*p*<0.01 versus HF+Lut (8μmol L-1), @*p*<0.05, @@*p*<0.05 versus HF+Lut (16μmol L-1), +*p*<0.05, ++*p*<0.05 versus HF+Lut (32μmol L-1).
